# Trastuzumab Resistance, a Potential Roadblock for Most Successful Therapy of Breast Cancer—An Updated Review of Underlying Mechanisms, Clinical Trials and Patents to Evade the Resistance

**DOI:** 10.3390/pharmaceutics18050514

**Published:** 2026-04-22

**Authors:** Gul Hasan, Soudipta Pramanik, Sandhya Singh, Pravin Gurav, Sudha Madhavi Penumaka, Sudheer Kumar, Debabrata Mandal

**Affiliations:** Department of Biotechnology, National Institute of Pharmaceutical Education and Research, Hajipur 844102, India; gulhasansaifi64@gmail.com (G.H.); pramanik.soudipta@gmail.com (S.P.); ss5469323@gmail.com (S.S.); pravingurav366@gmail.com (P.G.); madhavi47penumaka@gmail.com (S.M.P.); ksudheer093@gmail.com (S.K.)

**Keywords:** trastuzumab resistance, HER2 receptor, PI3K/AKT/mTOR pathway, Mucin1, combination therapy, patents & clinical trials

## Abstract

Trastuzumab is the first humanised monoclonal antibody (Mab) developed for breast cancer (BC) therapy. The high affinity of Trastuzumab Fab-domain binding to the human epidermal growth factor receptor 2 (HER2) receptor, with a K_d_ value of <1 nM, is also accompanied by Fc domain interaction with Fc-receptors in natural killer cells and leukocytes, enabling the killing of tumour cells through antibody-directed cellular cytotoxicity (ADCC). Trastuzumab blocks the over-expressed HER2 receptor-mediated dimerization and consequent intracellular signalling, leading to cancerous growth. However, the trastuzumab resistance (TR) became the major problem within 1 year of treatment. The mutation in phosphatidylinositol 3′-kinase (PI3K) pathway, cross-talk with estrogen receptors, over-expression of Mucin 1 (MUC1) protein, insulin-like growth factor I receptor, etc., are key pathways involved in TR. In this review, we have provided a molecular view of TR and the possible remedies for overcoming TR using BC stem cell (BCSC)-based therapy, PI3K pathway inhibitors, MUC1-based treatment, etc. We have also analysed the patents and clinical trials from the pre-TR and post-TR era to rationalise the possible steps to overcome TR. Our analysis implies that Trastuzumab monotherapy no longer applies to HER2+ BC treatment. Further, combination therapy using other antibodies like pertuzumab and protein kinase inhibitors and targeting pathways like the ubiquitin proteasome pathway will be the future option for BC Treatment. Overall, this review provides a detailed summary of the molecular mechanisms involving TR and its potential ways of evasion, based on updated information from published research articles, clinical trial outcomes, and patent data.

## 1. Introduction

In 2020, the International Agency for Research on Cancer (IARC), a specialised cancer agency of the World Health Organisation (WHO), released a report that showed that breast cancer (BC) has overtaken lung cancer as the most prevalent cancer throughout the globe [1]. BC is also the leading cause of cancer-related deaths in women worldwide [2]. The observed over-expression of HER2 in approximately 20–30% of BC patients has encouraged researchers for years to find a drug/antibody to target HER2 so that the drug can stop HER2-mediated BC. In 1984, gene ‘neu’ [3] was isolated from rat neuro/glioblastoma encoding a protein with 185 KDa, similar to a gene located at the human q21 region of chromosome 17 [4]. Due to the significant similarities of the ‘neu’ gene with the protein sequence of the EGFR (HER1), the ‘neu’ gene was named HER2, a kinase receptor at the cell surface. In 1986, it was proven for the first time that the NIH3T3 cells became tumorigenic when transfected with the HER2 gene in vivo [5]. Further, that study showed that treating the mice with an antibody against the ‘neu’ gene product (an anti-HER2 antibody) significantly suppresses tumour growth. In 1987, it was established that poor prognosis in BC patients is significantly correlated with the amplification of the HER2 gene in about 30% of BC tumours [6]. In 1989, a monoclonal antibody (Mab) “4D5” (based on the Hybridoma culture name) was isolated from BALB/c mice, which has a high specificity of binding toward human HER2 and not HER1; thereby selectively suppressing the progression of HER2-positive BC cells [7]. The mouse 4D5 monoclonal antibody was eventually humanized [8] and named Trastuzumab or Herceptin, which showed significant clinical efficacy toward patients with HER2-positive BC compared to conventional chemotherapies. In short, the discovery of a 185 kDa unknown transmembrane human receptor, now known as the HER2 receptor, inspired the development of the first humanised monoclonal antibody (Mab), Trastuzumab, which received FDA and EU approval in 1998 and 2000, respectively. The results from two clinical trials demonstrated the efficacy and safety of trastuzumab, where it was established that progression-free survival (PFS) was significantly longer due to the addition of trastuzumab to chemotherapy (7.6 vs. 4.6 months with chemotherapy alone), as was median overall survival (OS) (25.4 vs. 20.3 months) [9,10]. The success of trastuzumab and its subsequent commercialisation emerged from the work of 3 scientists: Axel Ullrich and Michael Shepard of Genentech, and Dennis Salmon of the University of California at Los Angeles (UCLA). Trastuzumab had 2018 worldwide sales of $7.5 billion. The patents on trastuzumab expired in the US in June 2019 and in Europe in July 2014. There are currently five trastuzumab biosimilars available in the USA. The global trastuzumab biosimilars market is expected to grow from $2.08 billion in 2021 to $2.64 billion in 2022 at a compound annual growth rate (CAGR) of 27.1%. These numbers indicate the importance of Trastuzumab in BC treatment.

The HER2 receptors, which are similar to epithelial growth factor receptor (EGFR, HER1), ErbB3 (HER3), and ErbB4 (HER4) consist of three main domains: (a) the extracellular domain that contains the four subdomains of HER2 receptor allowing ligand-dependent or -independent dimerization, (b) transmembrane domain that connects extracellular and cytoplasmic domains, and (c) the C-terminal cytoplasmic domain that contains tyrosine kinase and regulatory subdomains for activation of downstream signaling pathways [11]. To date, no HER2-specific ligand has been identified, indicating that HER2 can go for ligand-independent homo or hetero dimerization to activate the signaling pathway that leads to BC. Therefore, the inhibition of HER2 dimerization, the primary target of trastuzumab, leads to suppression of HER2-mediated cell signalling and tumour growth. One of the primary reasons for the particular binding of trastuzumab is that the Mab never has to compete with any intracellular ligand for binding with the HER2 receptor. However, during 2005–2007, it was observed that a fraction (~20%) of early-stage BC patients do not respond to trastuzumab, and ~70% of patients with metastatic disease who receive trastuzumab monotherapy are resistant to treatment within 1 year of treatment [12,13]. This indicates that although the application of anti-HER2-based trastuzumab represents a striking advantage over chemotherapy, resistance to trastuzumab therapy leads to recurrence, which limits the survival of HER2-positive BC patients. Therefore, the resistance of trastuzumab has become a global concern for BC treatment, considering the early success of monotherapy for this Mab. Therefore, this review initially focuses on the pathways related to TR. Further, we looked at different applications and strategies that dealt with overcoming this resistance. We have summarised the claims of different patents and clinical trials related to the pre- and post-TR era to identify a future direction for tackling this resistance.

## 2. Different Mechanisms of TR

### 2.1. HER2 Mutations

**Figure 1:**
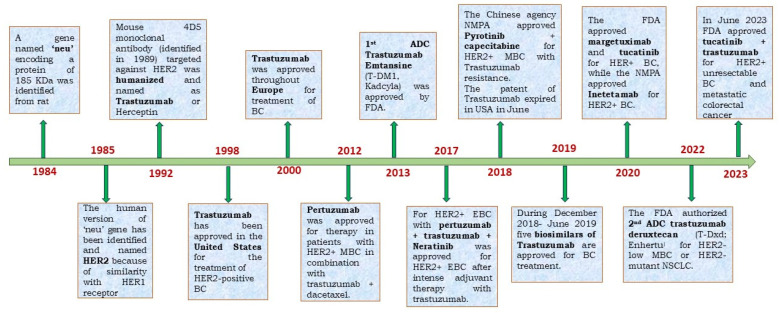
Timeline describing the brief History of the major developments of Trastuzumab (1994–2023) from gene discovery to ADC and its use for overcoming resistance.

The discovery of the HER2 receptor and its consequent overexpression in BC cells advanced the discovery of the first humanized monoclonal antibody, Trastuzumab, ADC, etc. (Figure 1). Trastuzumab cannot bind to the receptor when HER2 is mutated by deletion of its extracellular domain [14]. The shortened and N-terminally deleted p95HER2 isoform with constitutive kinase activity is produced by proteolysis or by translation of the HER2 mRNA from internal initiation codons [15,16,17]. This mutant isoform encourages ongoing oncogenic signalling activation while evading the effects of trastuzumab by not allowing any binding sites on the HER2 receptor for the mAb. Those who developed the p95HER2 mutation were less likely to respond to trastuzumab than those with full-length HER2. This was established in a study of 46 patients with metastatic BC tumours [17]. Among 46 patients with metastatic BC only, 11% of patients expressing p95HER2 responded to trastuzumab. However, >51% of patients with tumours expressing full-length HER2 achieved either a complete or partial response in the presence of trastuzumab. However, treatment with lapatinib (a tyrosine kinase inhibitor) shows almost equal response, indicating that tyrosine kinase activity is retained in p95HER2-expressing cells. The HER2 protein’s inability to bind trastuzumab is caused by several genetic abnormalities, which also contribute to TR. As an illustration, p95HER2, also referred to as the HER2 carboxy-terminal fragment, is deficient in the N-terminal extracellular domain required for trastuzumab binding. This fragment is produced either by the shedding of the extracellular domain by metalloprotease ADAM10 (also known as ‘sheddase’) or by the alternate start or translation of the HER2-coding mRNA. Through its capacity to constitutively create homodimers that are stabilized by disulfide linkages, p95HER2 enhances TR [18]. High levels of p95HER2 were found to be associated with shorter PFS and worse OS in metastatic BC patients receiving trastuzumab [19]. Therefore, for a fraction of HER2+ patients with metastatic BC, p95HER2 is an appropriate target.

### 2.2. Mechanism of TR Through Crosstalk Between HER2 and Estrogen Receptors

**Figure 2:**
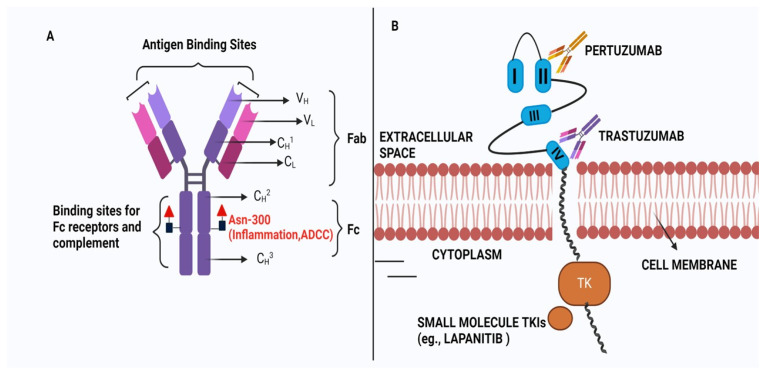
Schematic structure of Trastuzumab with Fab and Fc part, where residue important for Inflammation and ADCC is indicated (**A**). Specific binding domain of Trastuzumab, Pertuzumab and Tyrosine kinase inhibitors on HER-2 is indicated (**B**).

In 2006, Munzone et al. showed for the first time that a patient with HER2+ and ER-negative advanced BC can convert into an ER-positive patient after treatment with trastuzumab and chemotherapy [20]. It was concluded from the clinical trial data that inhibition of HER2 by trastuzumab and chemotherapy leads to the activation of ER gene transcription via signalling crosstalk pathways, involving the PI3K/AKT/mTOR pathway more prominently. This increased ER signalling pathway compensates for tumour growth and survival under trastuzumab treatment for HER2+ BC [21]. Consequently, there is a growing interest in combination treatments that simultaneously target both HER2 and ER (by endocrine therapy) to combat this resistance mechanism effectively [22]. However, a critical question remains whether patients who have previously received HER2-targeted therapies would benefit from continuing endocrine therapy alongside chemotherapy or Antibody–Drug Conjugate (ADC)-based treatments [23,24]. The phase II clinical trial (PERTAIN, NCT01491737) with patients of HER2+ and ER+ BC indicates that when ER is overexpressed, trastuzumab alone cannot work and combination therapy is required [25]. In this context, the trastuzumab and tamoxifen combined therapy was effective for HER2+ and ER+ BC. It was observed that patients with ER+ mBC showed better prognosis after treatment in combination with immunotherapy (pembrolizumab) and antiestrogen agents like letrozole or tamoxifen [26]. All these studies prove that targeting both HER2 and ER is more beneficial than HER2-targeted therapy alone. The mechanism of action of Trastuzuamb with its structure is shown in Figure 2.

### 2.3. Failure to Trigger ADCC

**Figure 3:**
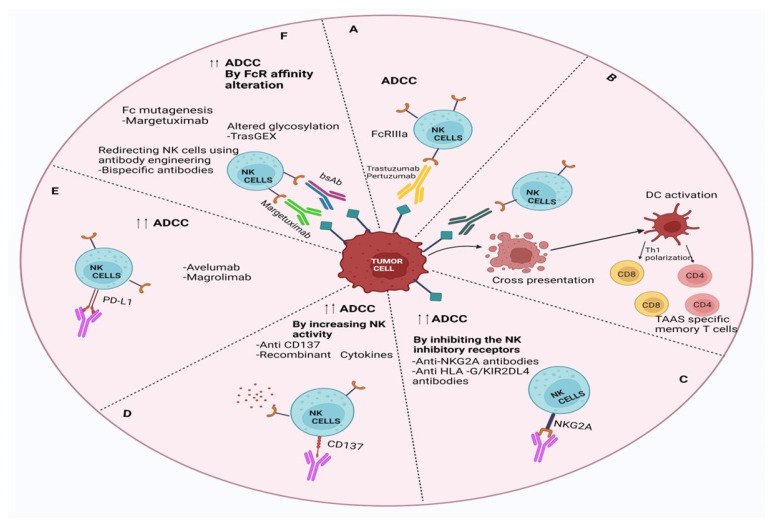
A schematic diagram of HER2-mediated therapy using Trastuzumab with a specific focus on ADCC.

The concept of immunomodulatory antibodies came into the limelight due to the discovery of cytotoxic T lymphocyte-associated antigen 4 (CTLA4) and programmed cell death protein 1 (PD1) as immune checkpoint inhibitors. The checkpoint proteins, like programmed death (PD)-1 (present in T cells), bind with PD ligand-1 (PD-L1), which is generally overexpressed in most cancer cells. This binding acts as an ‘off-signal’ for immune-mediated signalling, so cancer cells can evade T-cell-mediated death. A very similar mechanism is involved in ADCC, where the antibody’s constant region (Fc) interacts with Fcγ receptors (FcγRs), which recruit neutrophils and NK cells to the tumour cells, which are already bound by the Fab part of the antibody due to strong receptor-ligand interaction (Figure 3) [27]. The NK cells then release perforins and granules to kill the bound tumour cells by apoptosis. The initial observation of trastuzumab’s mechanism of action, involving ADCC, was first documented in preclinical animal models. Factors influencing the efficacy of HER2-directed therapy in ADCC induction include Tumour-Infiltrating Lymphocytes (TILs) and Fcγ receptor polymorphisms. The Fcγ receptors are abundant on leukocytes and recognise IgG-coated immune complexes through the Fc receptor of the antibody. Various strategies have been investigated to enhance ADCC in the context of HER2-targeted therapies. ADCC is governed by three Fcγ receptors expressed on immune cells: CD16A, CD32A, and CD32B [28]. To enhance ADCC, margetuximab (a chimeric Mab targeting HER2+ BC cells) was engineered to preserve the epitope specificity of trastuzumab while introducing five amino acid modifications, increasing its affinity for the activating Fcγ receptor IIIA (CD16A) and reducing its affinity for the inhibitory Fcγ receptor II (CD32B) [25,29]. This antibody is very similar to trastuzumab except that the Fc section is engineered to bind with both alleles of CD16A with improved ADCC. Due to progressive Trastuzumab treatment, the mutant version of allele CD16A-158V is generated in BC cells. This eventually makes Trastuzumab treatment ineffective for these BC cells due to reduced Fc-FcγRs interaction in ADCC [30]. In the phase 3 SOPHIA trials, margetuximab + chemotherapy and trastuzumab + chemotherapy were compared in 536 patients with previously treated HER2+ advanced BC+ metastatic BC (mBC). The PFS was much better for margetuximab + chemotherapy, even though HER2+ binding is similar for both margetuximab and trastuzumab. This indicates that better ADCC activity of margetuximab is responsible for a higher BC cure rate. In addition to failure in ADCC activity, antibody-dependent cellular phagocytosis (ADCP) mediated by macrophages also plays an important role in the development of trastuzumab resistance [31]. Under normal conditions, trastuzumab-coated HER2-positive tumour cells can be recognized and engulfed by macrophages through Fc receptor interactions, contributing to antitumor immunity [32]. However, during TR, this process becomes dysregulated due to changes within the tumour microenvironment (TME). Tumour-associated macrophages (TAMs) often undergo polarization toward an immunosuppressive M2 phenotype, which supports tumour growth, angiogenesis, and tissue remodelling rather than immune-mediated clearance [33]. These M2 macrophages exhibit reduced phagocytic activity against antibody-coated tumour cells and secrete anti-inflammatory cytokines such as IL-10 and TGF-β, further suppressing adaptive immune responses [34]. Moreover, overexpression of “do not eat me” signals, such as CD47, on tumor cells can inhibit ADCP by preventing macrophage recognition and engulfment [35]. Together, these alterations in macrophage function and signaling pathways contribute to an ineffective ADCP response, allowing HER2-positive tumour cells to evade immune-mediated elimination and promoting TR [36]. This highlights the importance of targeting macrophage polarization or blocking inhibitory pathways like CD47–SIRPα to restore effective ADCP and enhance the therapeutic efficacy of trastuzumab [37].

### 2.4. Changes in HER2 Downstream Signalling Pathways

#### 2.4.1. Loss of PTEN Expression/Function & P13K Mutation or Increased Activity

Humans produce an enzyme called Phosphatase and Tensin Homolog (PTEN), created by the PTEN gene [38]. This enzyme acts as a tumour suppressor by regulating cell division and preventing uncontrolled growth. Mutations in the PTEN gene are linked to various cancers, including BC. The PI3K/Akt is constitutively activated due to PTEN depletion [39]. Reduced PTEN expression or activity prevented trastuzumab-mediated growth suppression in BC cells overexpressing HER2 [40]. Numerous malignancies, including about 50% of BCs, have been shown to cause PTEN loss of function brought on by mutation or transcriptional regulation [41,42].

**Figure 4:**
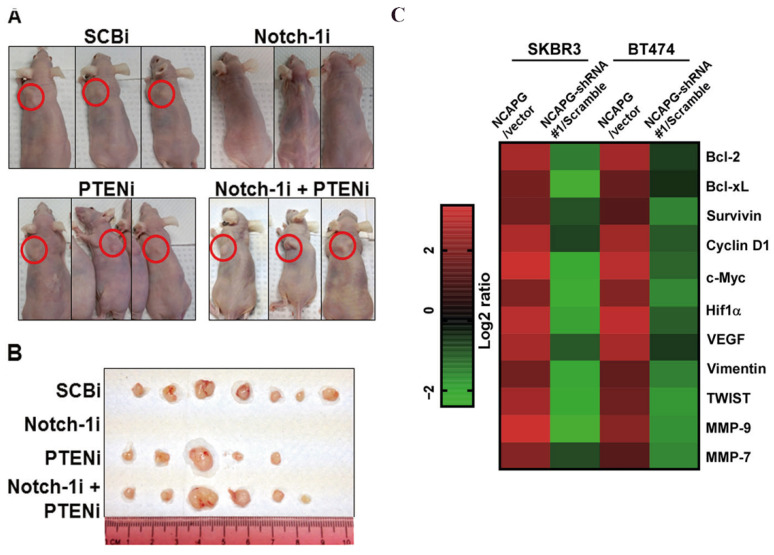
PTEN Signalling pathway inhibition by Notch 1 is required for trastuzumab-resistant tumour formation. (**A**) In the nude mice, it was shown that PTEN knockdown initiates tumour initiation, whereas Notch 1 knockdown inhibits tumour formation. (**B**) Tumour formation in the presence of different inhibitors, including Notch1. (**C**) mRNA expression of breast cancer cells promoted by non-structural maintenance of chromosome condensin 1 complex subunit G. (**A**,**B**) Reproduced/adapted with permission from [Nature] for reference [47]. (**C**) adapted with permission from Nature for reference [48].

Trastuzumab treatment outcomes were lower in patients with PTEN-deficient HER2-overexpressing MBC than those with normal PTEN tumours. Another study claimed that increasing Notch-1 expression in trastuzumab-resistant cells was required for tumour recurrence in vivo. They also demonstrated a mechanism by which Notch-1 is required for trastuzumab resistance by repressing PTEN expression to contribute to activation of ERK1/2 signalling. To determine if Notch-1-mediated inhibition of PTEN is required for trastuzumab-resistant tumour initiation, they tested the effect of Notch-1 and/or PTEN inhibition on the rate of tumour onset. PTEN knockdown alone resulted in tumour initiation at a similar rate to the control siRNA-transfected cells. Combined Notch-1 and PTEN knockdown resulted in a similar rate of tumour initiation compared to control, Notch-1, or PTEN siRNA [Figure 4]. These findings suggest that Notch-1 contributes to trastuzumab resistance by repressing PTEN, which may lead to hyperactivation of ERK1/2 signalling. TR has also been linked to oncogenic activating PI3K mutations. Berns and colleagues looked for activating PIK3CA mutations in hotspot areas of 55 HER2-amplified primary tumour samples from trastuzumab-refractory patients. According to earlier investigations on the frequency of mutations in HER2-amplified BC, 25% of tumours had mutations [43,44,45]. PTEN expression was also examined in this collection of cancers, and in 22% of tumours, decreased PTEN expression was seen. Patients with active PI3K (defined as decreased PTEN expression or PIK3CA mutation) exhibited a substantially lower PFS on trastuzumab-based treatment than patients without evidence of PI3K pathway activation, according to Kaplan–Meier survival curves. Further, PIK3CA (39%) and PTEN (20%) mutations were more common in cell lines than tumours, but PIK3CA mutations did not have a significant effect on clinical efficacy after adjuvant tamoxifen therapy in [46] HR + BC patients.

#### 2.4.2. Cyclin-Dependent Kinases and p27

In vivo, p27 can inhibit the kinase activities of a variety of cyclin/CDK complexes (where N-terminal domain is sufficient to block cyclin/CDK kinase activity) including those containing CDK1, -2, -4, or -6, and cyclins A, E, or D. Downregulation of the p27KIP1 protein is associated with poor prognosis in several human cancers, often due to its cytoplasmic localization or phosphorylation. Trastuzumab influences the CDK inhibitor p27Kip1, a key regulator of cell growth. It is crucial for cell cycle regulation by binding to the cyclin E-CDK2 complex during the G1 phase [49]. Proliferating cells exit the cell cycle when p27Kip1 levels rise, while quiescent cells resume proliferation when p27Kip1 levels decrease. Therefore, p27Kip1 plays a significant role in human cancer development due to its key function in cell cycle regulation [50].

Reducing cyclin E expression or using CDK2 inhibitors decreased cyclin E activity in trastuzumab-resistant clones, reducing proliferation and increasing apoptosis. These findings imply that cyclin E overexpression and CDK2 activity are directly involved in trastuzumab resistance, suggesting that CDK2 inhibitors could be an effective treatment for BC patients with co-amplification of HER2 and cyclin E. Trastuzumab inhibits HER2-overexpressing BC by inducing G1 cell cycle arrest, with an elevation of p27Kip1 and a decrease in CDK2. A post-translational modification of anti-HER2 antibodies controls the phosphorylation of the p27Kip1 protein, through which the protein is upregulated. The magnitude of G1 cell cycle arrest induced by trastuzumab or 4D5 is well correlated with the induced level of p27Kip1. Anti-HER2 antibody-induced p27Kip1 protein, G1 arrest, and growth inhibition persist at least 5 days after 14 h of treatment with a single dose of trastuzumab [51].

#### 2.4.3. Role of Epigenetic Modifications in Trastuzumab Resistance

##### Histone Modification

Cancer cells contain altered post-translational histone modifications, including the loss of specific histone acetylation and methylation, in human carcinomas. For example, specific histone lysine acetylation (H3K9ac, H4K12ac, H3K18ac, and H4K16ac), lysine methylation (H3K4me2 and H4K20me3), and arginine methylation (H4R3me2) were identified using immunohistochemistry studies in human breast carcinomas of a significant population (n = 880) [52]. Histone acetylation (H3K9ac, H4K12ac, H3K18ac, and H4K16ac) and di-methylation at H3K4 (H3K4me2) are examples of activating histones that are frequently enriched at the ERBB2 promoter and enhancer regions in trastuzumab-sensitive tumours. This promotes an open chromatin conformation and high ERBB2 transcriptional activity. The chromatin activity of the ERBB2 locus controls the expression of the ERBB2 gene in BC. Because their ERBB2 chromatin is more open and active, these BC cells have greater levels of ERBB2 expression [53].

##### DNA Methylation

DNA methylation is a reversible epigenetic process in which methyl groups are added to the fifth carbon of cytosine residues using S-adenosyl methionine (SAM) as the methyl donor. This reaction is catalyzed by DNA methyltransferases (DNMTs), primarily DNMT1, DNMT3A, and DNMT3B. Here, DNMT1 primarily functions as a maintenance methyltransferase that preserves methylation patterns during DNA replication by methylating hemi-methylated DNA [54]. Whole-genome analyses have demonstrated a strong correlation between DNA methylation patterns and BC pathogenesis, identifying 345 methylated genes across 40 BC cell lines. Palomeras et al. looked into how DNA methylation contributes to TR in BC that is HER2-positive. Up to 62% of individuals experience resistance to trastuzumab after a year, despite the drug’s initial potent therapeutic effects. Researchers analysed the DNA methylation patterns of trastuzumab-sensitive (SK) and resistant (SKTR) cell lines using Infinium Human Methylation 450K arrays, taking into account methylation variations that were greater than 60%. The expression and methylation of five potential genes—TGFβ1, CXCL2, SLC38A1, and NR2F2—consistently changed across SK and SKTR cell lines. Methylation-specific PCR, qPCR, and treatment with the demethylating reagent 5-aza-DC were used to validate these results. According to this study, TR in individuals with HER2-positive BC may be predicted or monitored by hypermethylation of these five genes [55].

In another study, hypermethylation and downregulation of the TGF-Β1, CXCL2, and SLC38A1 genes were observed in trastuzumab-resistant SKTR cell lines. These results were validated by qRT-PCR and bisulfite pyrosequencing, with TGFβI exhibiting the highest association between transcriptional suppression and promoter hypermethylation [56]. Here, restoring TGF-βI expression in resistant cells improved trastuzumab sensitivity. Additionally, TGF-βI hypermethylation was found to be substantially linked to a poor response to trastuzumab-based therapy in patient samples with HER2+ BC, implying its potential as an early indicator for TR [56].

##### MicroRNAs (miRNAs)

MicroRNAs (miRNAs) are non-coding RNAs of 18–25 nucleotides in length that prevent the expression of genes by binding to or degrading them. To date, more than 1000 distinct miRNA genes [57] have been discovered, which regulate gene expression. Thirty per cent of human genes are thought to be targeted by miRNAs’ interactions with various genes, which are engaged in a wide range of biological processes, such as the cell cycle, cancer, metabolic regulation, immunological response, and apoptosis [58]. It has been found that several miRNAs are expressed abnormally in HER2-positive BC cell lines and are linked to TR. The increased production of miR-21 prevents apoptosis in cancer cells, promoting their growth. Higher expression of miR-21 is inversely linked with PTEN expression, and loss of PTEN causes resistance to trastuzumab via activating the PI3K pathway [59]. Between trastuzumab-resistant and -sensitive cell lines, microarray analysis identified 151 substantially different miRNAs, including 46 upregulated and 105 downregulated miRNAs. QRT-PCR validated seven miRNAs with consistent expression patterns. According to functional enrichment analysis, the biological processes associated with trastuzumab resistance are primarily regulated by the PI3K-Akt signalling pathway. A study of patients’ sera revealed that cases of trastuzumab resistance were associated with significantly downregulated miR-224 and increased expression of miR-200b, miR-135b, and miR-29a. Therefore, these four miRNAs were shown to be highly linked with trastuzumab resistance, indicating their potential as predictive biomarkers. The protein–protein interaction (PPI) network also revealed three subnetwork modules that shed more light on the molecular processes behind trastuzumab resistance involving these miRNAs [60].

Rezaei et al. proposed a possible correlation between plasma miRNAs and trastuzumab response in patients with HER2-positive BC. According to their findings, resistant BT-474 cells have considerably higher levels of miR-1246 and miR-23b-3p and lower levels of miR-195-5p and miR-34c-3p. The resistant group had significantly lower levels of miR-195-5p, miR-34c-3p, and miR-1246 in plasma samples, but there was no statistically significant difference in the expression of miR-23b-3p. Furthermore, the expression of c-MET, CCND1, MAP2K1, and PTEN was significantly elevated in these samples. These findings suggested that miR-195-5p, miR-34c-3p, and miR-1246 might be potential biomarkers for prognosis and early identification of the trastuzumab-resistant population from the sensitive group of patients with HER2-positive BC [61].

### 2.5. Overexpression of Other Receptors and Proteins in Resistance

#### 2.5.1. Increased Expression of IGF-1R

The insulin-like growth factor 1 receptor (IGF-1R) may play a role in TR. Lu et al. found that excessive IGF-1R levels can make trastuzumab-sensitive SKBR3 cells resistant to the therapy. These IGF-1R-overexpressing cells have reduced p27Kip1 and p21Cip1 proteins and increased CDK2 kinase activity [62]. Jerome et al. proved that IGF1R-overexpressing cells play a role in developing TR by escaping cell cycle arrest caused by trastuzumab. This study evaluated human IGF binding protein 3 (rhIGFBP-3), an antagonist of IGF-IR signalling, in Trastuzumab-resistant BC cells in vitro and in vivo. It was observed that rhIGFBP-3 decreased IGF-I-induced IGF-IR phosphorylation and worked synergistically with Trastuzumab against HER-2-overexpressing BC cells in vitro. Furthermore, a high degree of IGF-1R expression in HER2-amplified cell lines is associated with a reduced response to trastuzumab [63,64]. This is due to HER2 and IGF-1R interaction, with IGF-1 activation resulting in HER2 phosphorylation and PI3K stimulation. IGF-1R signalling attenuation stops HER2 phosphorylation and restoration of trastuzumab sensitivity [65]. These results provide an opportunity to evaluate the simultaneous blockade of the HER-2 and IGF-IR pathways using a combination therapy, which may include rhIGFBP-3 and trastuzumab, in patients with HER-2-positive BC.

#### 2.5.2. Increased Activity of Rac1 and Activation of TNFα

The Rho family of GTPases is a family of small (~21 kDa) monomeric G proteins that belong to the Ras superfamily of GTPases. The Ras superfamily of GTPases comprises more than 50 members with several common features: their molecular weight (18–28 kDa), their C-terminal poly-isoprenylation region, and the property to bind to and hydrolyse guanine nucleotides. The Rho GTPases form 8 subfamilies, among which one subfamily comprises Rac1, Rac2, Rac3, and RhoG. In SKBR3 cells, resistance to trastuzumab was linked to elevated Rac1 activity, which resulted in considerable cytoskeleton disarray. Decreased extracellular signalling regulates kinase function in resistant clones, trastuzumab-facilitated endocytic downregulation of HER2, and low Rac1 activity [66]. Further, it was shown that dysregulation of classical Rho GTPases like Rho, Rac, and Cdc42 can initiate BC and its metastasis [67].

Tumour necrosis factor-α (TNFα) activates c-Src to cause HER2 phosphorylation in BC cells. TNFα-induced NFkB activation and cell proliferation were inhibited by HER2 knockdown via RNA modification or by the use of AG825, an inhibitor of EGFR/HER2-TK. Trastuzumab, nevertheless, was unable to prevent TNFα from stimulating the growth of BC. It is interesting to note that TNFα can trans-express HER2 and employ it as a downstream signalling molecule necessary for mitogenic signalling production. Since many people with breast tumours have TNFα in their tumour microenvironment, treating HER2-positive patients with TNF-α as a target may be possible [68,69]. The blockade of TNF-α was proposed as a therapy to treat BC since TNFα enhances luminal BC cell proliferation by aromatase upregulation. This aromatase enzyme increases estradiol synthesis, which, after binding to ER, promotes luminal cancer cell proliferation [70]. Stable overexpression of TNFα in BT-474 cells, a human HER2-positive BC cell line, led to high activation of the Akt and NF-κB signalling pathways. Further, it was proven that TNFα-induced expression of mucin 4 is partially responsible for TR in HER2-positive BC [71].

## 3. Different Ways of Overcoming Trastuzumab Resistance

### 3.1. BC Stem Cell (BCSC)-Specific Therapeutic Approaches for TR

The aggressive progression and recurrence of BC were due to the presence of a subset of tumour cells known as BC stem cells (BCSCs). These cells possess the abilities of self-renewal and tumour initiation, which allows them to drive metastases and tumour growth. These cells reside in a microenvironment filled with resident inflammatory cells that generate signalling cues for BCSC-mediated self-renewal and survival [72].

Three key proteins were known markers for BCSCs: CD44, CD24, and aldehyde dehydrogenase (ALDH). However, studies have shown that, sometimes, BCSCs contain both CD44 and ALDH, but not CD24 [73]. The behaviour of BCSCs is regulated by several pathways, including PI3K/AKT, TGF-/SMAD, Wnt/Catenin, Notch, JAK/STAT, NF-B, and other signalling pathways. Therefore, crosstalk or deregulation of those pathways may be a factor in the growth or transition of BCSCs in controlling TR.

#### 3.1.1. Therapeutic Approaches Targeting Different Signalling Pathways

**Figure 5:**
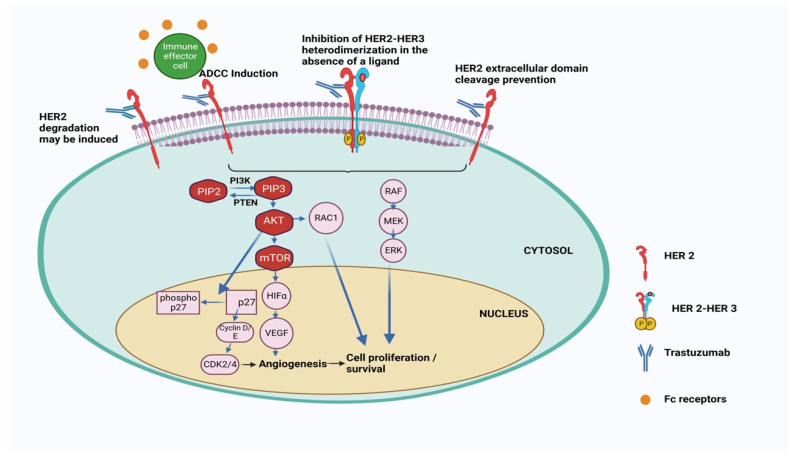
A schematic presentation of the Trastuzumab resistance mechanism involving different signalling pathways, where the PI3K/AKT/mTOR pathway is highlighted.

Dysregulation of the PI3K/AKT pathway and loss of the tumour suppressor Phosphatase and TENsin homolog (PTEN), a downstream mediator of the PI3K/AKT pathway [74,75], have been linked to adaptation or resistance to trastuzumab and have been found in over 40% of HER2-positive BCs [76]. PTEN loss may cause TR in HER2-positive BC by initiating epithelial-mesenchymal transition (EMT) and switching the CD44+/CD24−/low/HER2−/low subtypes. The dimerization of HER2 with its coreceptor HER3 and subsequent direct coupling to the p85 regulatory subunit of PI3K and activation of PI3K-AKT signalling, which drives ALDH+ populations in HER2-overexpressing cells, is another explanation for the emergence of TR [77]. Trastuzumab could decrease the ALDH+ population but not eradicate it [78,79]. As a result, PI3K inhibitors might be able to lower the proportion of BCSCs (including CD44+/CD24/low and ALDH+ cells), hence lowering the HER2-positive BC susceptibility to trastuzumab [80,81]. When coupled with trastuzumab, the pan-PI3K inhibitor, XL147 lowered BCSC fractions and slowed the carcinogenesis, possibly by preventing acquired TR [82]. Additionally, an AKT inhibitor reduced the PI3K/AKT pathway and helped HER2-positive BC patients’ refractory BCSCs regain their sensitivity to trastuzumab. The mechanism of Trastuzumab resistance involving different signalling pathways is shown in Figure 5.

One study found that inhibiting TGF-β signalling, which effectively controls the EMT and the maintenance of BCSCs, reduced resistance to trastuzumab [83]. A83-01, a different small molecule inhibitor, could alter the TGF-β/SMAD pathway, thereby stopping HER2-positive cells from developing a mesenchymal phenotype and improving the HER2-positive cells’ response to trastuzumab therapy [84]. The Wnt3/β -catenin signalling activation has been shown to drive resistance to endocrine treatment, chemotherapy, and radiotherapy in BCSCs [85,86]. The expression of Wnt3 in trastuzumab-resistant cells is correlated with increased nuclear expression of β-catenin and transactivates the expression of EGFR. The increased Wnt3 in the TR cells also promoted a partial EMT-like transition, like increased N-cadherin, Twist, and Slug expression and decreased E-cadherin expression., This was associated with the emergence of TR in HER2-overexpressing BC cells. Conversely, knockdown of Wnt3 by siRNA restored the cytoplasmic expression of β-catenin and decreased EGFR expression in trastuzumab-resistant cells. The Wnt3 transfectants of Trastuzumab-resistant cells, SKBR3/Wnt3-7 and SKBR3/Wnt3-9, showed a significant decrease in E-cadherin and an increase in N-cadherin, Twist, and Slug. Therefore, increased Wnt3 expression and associated β-catenin signalling lead to TR.

**Figure 6:**
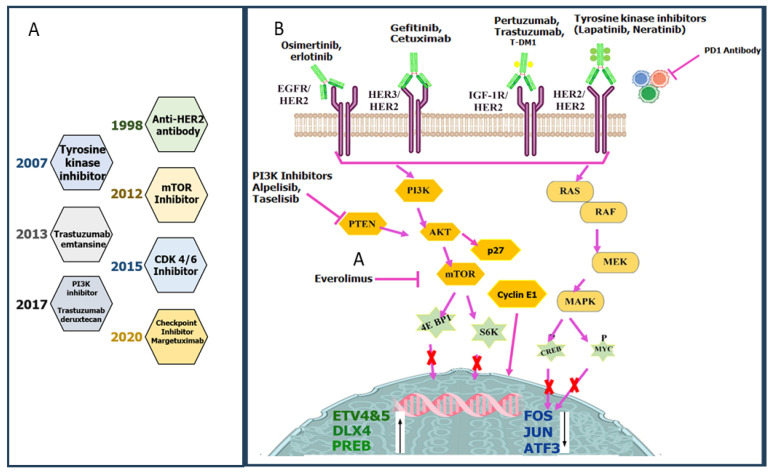
A timeline of FDA approval of the development of small molecule inhibitors for the therapy of BC in combination with Trastuzumab and its ADC (**A**), and A schematic presentation of inhibitors targeting different pathways to overcome Trastuzumab resistance (**B**).

The mesenchymal CD44+/CD24/low phenotype in HER2-overexpressing BC was strongly related to TR and HER2 overexpression-activated STAT3, which causes upregulated CD44 expression. According to reports, the STAT3 inhibitor static kills the CSC phenotype in HER2-positive breast tumours, suggesting that JAK/STAT signalling may be a potential target for trastuzumab-resistant BC [87]. Further, combined treatment of Herceptin and Stattic showed a synergistic effect on the cancer cell growth in vitro. When the STAT3 gene was knocked down, the expression of the stem cell markers (OCT-4, SOX-2, and CD44) was downregulated, and tumour formation was abolished. It was concluded from the study that targeting STAT3 may overcome Trastuzumab-induced resistance in HER2-overexpressing breast tumours. Jiang, Lili et al. showed that non-structural maintenance of chromosome condensin 1 complex subunit G (NCAPG) expression was highly upregulated in trastuzumab-resistant HER2+ BC. Figure 4C shows that NCAPG promotes several STAT3 downstream genes. So, knockdown of the NCAPG can reverse the trastuzumab resistance [48]. Figure 6 represents the proposed inhibitors and their related pathways to overcome Trastuzumab Resistance.

**Figure 7:**
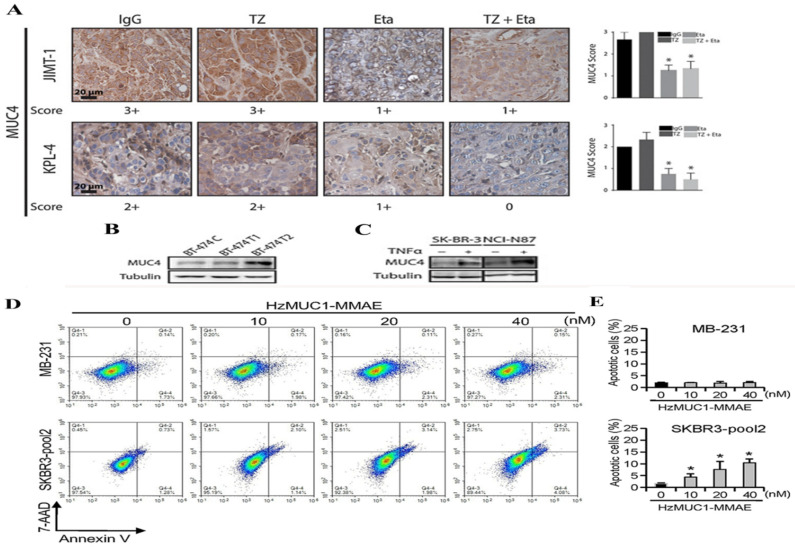
TNFα induces MUC4 expression in vivo and in vitro in BC cells, showing the importance of MUC4 in BC. (**A**) IHC of MUC4 expression decreased in the presence of TNFα inhibitor. (**B**,**C**) MUC4 levels were checked by Western blot in the presence of TNFα in TR cells (**D**). HzMUC1-MMAE induces apoptosis in HER2+ BC cells. (**E**) Quantification of the flow cytometry analysis data from (**D**). (**A**,**C**) Reproduced/adapted with permission from [AACR] for reference [71]. (**D**,**E**) Reproduced/adapted with permission from [SciEngine] for reference [89], * *p* < 0.05.

Mucin-4 (MUC4), a highly O-glycosylated membrane protein also thought to be HER2’s partner, was associated with a poor prognosis in BC and other carcinoma types [88]. The study using Trastuzumab-resistant JIMT-1 cell lines showed that MUC4 is overexpressed. Further knockdown of MUC4 at RNA levels increases trastuzumab susceptibility. In another study, TNFα-induced MUC4 expression was proposed as a novel TR mechanism where the administration of TNFα-blocking antibody etanercept with trastuzumab downregulated MUC4 [71]. It was also confirmed by TNF α treatment in BT474 and other HER2-overexpressing BC cell lines [Figure 7].

Therefore, steric Inhibition of Antibody–Receptor Interaction in overexpressed MUC4 is a possible mechanism of TR and TNFα signalling involved in TR [90]. The mBC cell line developed TR by upregulating MUC1*, a cleaved version of the MUC1 protein, where treatment with MUC1 antagonists can overcome the resistance [91]. Further, the humanized antibody targeting MUC1 reduced the growth of HCC1954 xenograft tumors by inhibiting cell proliferation, thereby reducing TR [89]. Wu, G. et al. also showed apoptosis induction by flow cytometry assay in the HER2-positive cancer cell line by humanized MUC1 treatment [Figure 6]. It was also found that MUC1 silencing reduces NF-κB activity in cancer cells, interrupting the self-renewal of tumour cells. Therefore, TR have a direct correlation with overexpressed MUC4 and MUC1 expression.

Conventional targeted therapies align well with the Goldie and Coldman mathematical model, which emphasizes early intervention to prevent resistance and disease progression. Recent advances in genetics and molecular biology have reshaped treatment strategies—not only focusing on primary tumour removal but also on disrupting the cancer cell homing process. These advancements aim to prevent the formation of premetastatic niches and cancer-associated fibroblasts [92]. Such outcomes can be achieved through cyclically administered immunotherapy, either in conjunction with adjuvant hormone therapy or with a limited number of chemotherapy cycles. In a recent study on HER2^+^ gastric cancer, it was found that trastuzumab resistance can be acquired through the epithelial–mesenchymal transition (EMT) and the endoplasmic reticulum–associated degradation (ERAD) pathway [93]. The study also identified tumour HLA loss as a significant mechanism underlying resistance to T-DXd. Another contributing factor is the immune-cold phenotype in patients with high ERBB2 expression, determined by spatial profiling. Previously in this study, we have discussed different mechanisms responsible for trastuzumab resistance. Upon examining these mechanisms and other signalling pathways, such as PI3K/AKT/mTOR, AMPK, and HIF1α, it becomes clear that they are a consequence of metabolic reprogramming. Resistant tumour cells remodel their metabolism and enhance glycolysis, increase reliance on oxidative phosphorylation (OXPHOS), and up-regulate lipid and fatty acid metabolism to sustain growth and survival despite HER2 blockade [94].

Additionally, mitochondrial biogenesis, redox adaptation through ROS modulation, and glutamine dependence further contribute to cellular persistence under therapeutic stress. In a separate study, it was found that EbRb2, a 6-phosphofructo-2-kinase/fructose-2,6-bisphosphatase three inhibitor [95], reduces glycolysis and helps kill resistant breast cancer cells. It was also found that glutamine is a significant source of carbon and nitrogen for the cancer cells. Recent studies have shown that glutamine uptake inhibitors, GLS inhibitors, and glutamine-mimetic antimetabolites with immune checkpoint blockers synergistically affect cancer cell growth. Using different metabolic biomarkers to predict trastuzumab resistance and integrating this information with metabolic phenotyping, future personalised treatment strategies can be planned.

#### 3.1.2. Role of Cytokines, Immune Modulators and Modified Tumour Microenvironment

The IL-6 and IL-8 levels were considerably increased in BCSCs that were enriched during long-term trastuzumab treatment. An important approach that could be utilized to combat TR is the inhibition of the IL-6 inflammatory feedback loop and the reduction in the BCSC population, particularly in the CD44^+/^CD24^/low^ subgroups [96]. When Trastuzumab was combined with SCH563705, an inhibitor of the chemokine (C-X-C motif) receptor 1/2 (CXCR1/2), the treatment effectively eliminated BCSC-like cells. These results suggested that HER2-positive BCSCs can be targeted effectively by combining CXCR1/2 and HER2 signalling [96]. PD-L1 is a crucial negative regulator that aids different cancer cell types in evading the immune response. It was found that via stimulating the PI3K/AKT signaling pathway, PD-L1 had a significant impact on maintaining the stemness of BC cells. Recent findings indicate that utilizing immunotherapy against PD-L1 may be beneficial for treating HER2-overexpressing human BC cells cocultured with peripheral blood after trastuzumab treatment [97]. It was concluded that Trastuzumab-mediated upregulation of PD-L1 through engagement of immune effector cells may function as a possible mechanism of TR. It indicates that adding anti-PD-1 or anti-PD-L1 therapy to trastuzumab-based treatment may be a good alternative for overcoming TR against HER2+ BCs.

Strategies that target the BCSC tumour microenvironment (TME) may be effective since, as was already indicated, it plays a role in the emergence of treatment resistance. Tranilast (the TGF-β inhibitor), when combined with Doxil nanomedicine, improves the TME in a mouse model of TNBC is improved with increased perfusion and oxygenation [98]. This contributes to enhanced anti-tumour immunity with anti-PD-1/anti-CTLA-4 antibodies. BCSCs are more prevalent in hypoxic conditions where an oxygen-deprived environment is crucial to their proliferation, stemness, and self-renewal. AzCDF, a small molecule, was reported by Kim et al. [99] to target BCSCs in a hypoxic environment, thereby inhibiting tumour growth and reducing tumorigenesis. By reducing the number of BCSCs and preventing cancer cell metastasis, the suppression of TGF-inducible protein expression decreased hypoxia and tumor angiogenesis [100].

## 4. MUC1 Is a Possible Target to Overcome TR

### 4.1. Strategies for Mucin Targeting to Overcome TR

MUC1 is a member of the mucin family and plays oncogenic/mitogenic activities in cancer cells, interacting with multiple other oncogenic receptors and pathways, including HER2, β-catenin, NF-kB, and the estrogen receptor (ER). Furthermore, it has been proven that the MUC1 C-terminal domain (MUC1-C) promotes the development of TR and that silencing the MUC1-C proto-oncogene is related to greater susceptibility of HER2+ cells to trastuzumab-induced inhibitors of growth.

Research findings have shown that MUC1 is an excellent antigen for immunotherapy. Firstly, it is found on the surface of a breast tumour. Secondly, malignant MUC1 is hypo-glycosylated, indicating that its core antigen is accessible [101]. Thirdly, malignant MUC1 differs from normal MUC1, which is exclusively overexpressed in cancer cells. The fourth important information is that the MUC1 peptide sequence (PDTRP) is one of the most immunogenic MUC1 epitopes, which is recognized by the SM3mAb, which is an antibody against stripped MUC1 core protein. MUC1 is a good target for mAb, vaccines, and inhibitors because of this epitope [102]. Using anti-MUC1 mAb or siRNA of MUC1 targeting the MUC1 gene would render BC cells vulnerable to trastuzumab-mediated ADCC [103]. The strategies for MUC1 targeting in BC therapy are discussed below in detail. These include both immunotherapeutic and non-immunotherapeutic methods.

### 4.2. MUC1-Based Therapy Using Mab, Vaccines and CAR T Cells

The anti-MUC1 antibody AS1402 is evaluated in patients with metastatic BC in the Phase-I clinical trial. These individuals were previously treated, developed anthracycline or taxol resistance, and were able to tolerate this antibody [104]. Additionally, researchers have created an anti-MUC1 scFv (single-chain Fragment variable), also known as nanobodies, that bind to BC cells that express MUC1 and prevent their invasion and survival [105]. It was observed that the cytoplasmic domain of MUC1 (MUC1-C) accelerates the development of resistance to trastuzumab, and therefore silencing MUC1-C proto-oncogene is associated with increased sensitivity of HER2+ cells to trastuzumab treatment. In a mouse model of stage IV human BC, a flagella vaccination that targets MUC1 has been tried to decrease metastasis in these animals [106]. Additionally, the clinical trial of PANVAC, a MUC1 and carcinoembryonic antigen (CEA)vaccine, on patients with metastatic BC showed promising results [107]. In the clinical environment, L-BLP25, a peptide vaccine that targets MUC1 and CEA, is being tested on BC patients [108]. After the trial, core biopsies of patients were evaluated for MUC1 by immunohistochemistry (IHC; N = 691) and quantitative RT-PCR. High MUC1 protein and mRNA expression were correlated with a lower probability of pathologic complete response and with longer patient survival. Chimeric antigen receptor (CAR) T cells are engineered T cells that have a transmembrane domain, a signaling domain, and scFv from a particular tumour Ag-Antibody [109]. Targeting MUC1 in cancer has also been done using CAR T cell settings.

## 5. Overcoming Trastuzumab or HER2-Mediated Therapy via the PI3K/AKT/mTOR Pathway

Resistance to HER2-targeted therapy may emerge due to abnormal activation of signalling pathways downstream of the receptor [110]. Since HER2 mediates signal transduction via the PI3K/Akt/mTOR pathway, inhibiting components of this pathway may be a feasible strategy to overcome resistance and restore sensitivity to HER2-targeted therapy [111]. The abnormal regulation of PI3K/Akt signalling leads to upregulation of the downstream mTOR pathway, increased mRNA translation, and raised cellular proliferation [112,113], which is mediated by growth factor receptor amplification and loss of the PTEN tumour suppressor [114]. BC models with active PI3K/Akt/mTOR pathways demonstrate resistance to HER2-targeted therapies [115], and hyperactivity of the pathway related to BC involves gain-of-function mutations in genes encoding PIK3CA, which encodes the catalytic subunit of PI3K, along with mutations in AKT1 [116]. PTEN was discovered as the major gene out of 8000 evaluated genes whose suppression resulted in TR. The study reveals that in a cohort of 55 BC patients, activation of the PI3K pathway, which is based on the presence of oncogenic PIK3CA mutations or low PTEN expression, was associated with poor prognosis after trastuzumab therapy. Trastuzumab, combined with everolimus, an mTOR inhibitor, inhibited the growth of trastuzumab-resistant cells [117]. mTOR is a serine/threonine kinase that controls the signalling pathway of growth factors and hormones, thereby controlling cell growth and angiogenesis. Further, combining therapy with an mTOR inhibitor and trastuzumab was more effective in inhibiting tumour growth than monotherapy [118]. Other mTOR, PI3K, and dual PI3K/mTOR inhibitors, including NVP-BKM120, GDC-0941, NVP-BEZ235, and INK-128, have restored sensitivity to HER2-targeted therapy in cell culture-based and in vivo models of BC [119]. A phase 1b dose-escalation study of everolimus, trastuzumab, and paclitaxel involved 33 patients with trastuzumab-resistant metastatic HER2+ breast cancer. The therapy reported an overall response rate (ORR) of 44%, a disease control rate of 74%, and a median PFS of 34 weeks, much better than monotherapy of trastuzumab [120]. The combination of HER2-targeted therapies and BAY 80-6946 (a PI3K inhibitor) inhibited BC growth more effectively than either monotherapy. The combination restores sensitivity to trastuzumab and lapatinib in cells resistant to either drug [121].

## 6. RAS/RAF/MEK/ERK Pathway Related to Breast Cancer

The RAS/RAF/MEK/ERK pathway is linked to fundamental functions, including cell survival and proliferation, which are often altered in malignancies. Drugs that target molecules implicated in this pathway have not yet proven successful in clinical trials for treating breast cancer. While they have shown promise in in vitro studies, the RAS/RAF/MEK/ERK pathway is often activated in breast cancers, suggesting its significance in tumour progression [122].

Hasegawa et al. showed that the Ras/Raf/MEK/ERK or PI3K/Akt/mTOR pathway is the main downstream signaling pathway inhibited by trastuzumab resistance in HER-positive cancer cells, which are associated with the activation of downstream signaling pathways involving PI3K/AKT/mTOR and Ras/Raf/MEK/ERK. Elevated IGF-1R signaling also leads to trastuzumab resistance by activating the Ras/Raf/MEK/ERK and PI3K/Akt/mTOR pathways [14,40]. According to their findings, miR-205 expression decreases via HER2-downstream Ras/Raf/MEK/ERK signaling, which contributes to trastuzumab resistance. Inhibitors of the Ras/Raf/MEK/ERK pathway were found to upregulate miR-205 in MDA-MB-453 cells with HER2 amplification [123,124]. Here, miR-205 expression in HER2-overexpressing BC cells was significantly increased by treatment with MEK inhibitor, Raf-1 inhibitor, and ERK inhibitor but not with PI3K inhibitor and p38 MAPK inhibitor. This indicates a specific role ERK/MEK pathway in TR. The HER2 signalling via the Ras/Raf/MEK/ERK pathway causes promoter hypermethylation by activating the DNMT family proteins. This finding provides new insights into the signalling system that regulates miR-205 in breast cancer through the Ras/Raf/MEK/ERK pathway [123,124].

## 7. Trastuzumab Resistance and Role of CD74-Akt Signaling Axis

CD74 is a protein that acts as a chaperone for MHC class II molecules, helping in antigen presentation. Further, it also acts as a receptor for macrophage migration inhibitory factor (MIF), a cytokine that regulates immune responses. The binding of MIF to CD74 activates several signalling pathways, including the AKT signalling pathway, especially in TNBC. Therefore, these signalling effects on cell survival, proliferation, and inflammation are only related to TNBC and not HER2-positive BC. In short, the inhibition of Akt signaling, which promotes BC, reduces the CD74 expression, thereby reducing CD74-MIF interaction. Therefore, the CD74-AKT signalling axis is not directly related to TR or HER2-positive BC therapy. In a study, it was identified that the CD74/AKT signalling axis mediates drug resistance in TNBC and cancer stem cells by inhibiting AKT, which in turn disrupts NF-κB–Bcl-2 survival signalling, leading to decreased caspase activation and apoptosis of cancer cells. AKT inhibitors can inhibit the binding of phosphorylated AKT (with serine 473) to CD74. Combining AKT inhibitors with CD74-derived peptides further enhanced the apoptosis of cancer cells, suggesting a potential therapeutic strategy for chemo-resistant TNBC [125].

## 8. Trastuzumab Resistance in Contrast to Tyrosine Kinase Inhibitor Resistance

Trastuzumab resistance and tyrosine kinase inhibitor (TKI) resistance in HER2-positive BCs exhibit distinct mechanistic differences that reflect their different modes of action [126]. For this reason, the combination therapy including Trastuzumab and different TKIs is frequently used for the treatment of HER2-positive BC. Resistance to TKIs, such as lapatinib, neratinib, and tucatinib, commonly involves the acquisition of specific HER2 kinase domain mutations, most notably the L755S mutation, which reactivates HER2 signalling despite the presence of TKIs [127]. The L755S mutation activates the HER2 protein even in the presence of TKIs, resulting in active signalling through the MAPK and PI3K/AKT/mTOR pathways. The activated HER2 does not bind to reversible TKIs, such as lapatinib, but irreversible TKIs, like neratinib, show some binding efficacy. Significantly, lapatinib and neratinib resistance is associated with HER2 L755S mutation combined with PIK3CA mutations, while tucatinib resistance involves EGFR amplification and heterodimerization with amplified EGFR [128]. Furthermore, TKI resistance mechanisms include the upregulation of conventional drug efflux pumps (ABCB1, ABCC1), which actively efflux small molecule drugs out of cells—a mechanism not relevant for the large antibody, such as trastuzumab [129,130]. Cross-resistance patterns also differ: while lapatinib and neratinib resistance confer cross-resistance to tucatinib due to shared HER2 mutations, these resistant cells may remain sensitive to antibody-based therapies, such as Trastuzumab or T-DM1 [128].

While both resistance types share some common downstream pathway alterations, such as PI3K/AKT hyperactivation and metabolic reprogramming, the molecular basis differs significantly. The trastuzumab resistance primarily involves receptor-level changes and immune evasion, whereas TKI resistance involves kinase domain alterations/mutations and drug metabolism modifications [127,131]. These distinct mechanisms suggest that combination strategies targeting both the receptor expression and downstream pathways, using antibody-based and TKI-based therapies, may help to overcome resistance and improve patient outcomes [128,132].

## 9. Patents and Clinical Trials of Pre-Trastuzumab Resistance Period

The innovations in antibody engineering (US5821337A), alternate production and diagnostics (US5750176A) advanced the trastuzumab’s efficacy and safety profile. For example, the patent US5821337A, filed by Genentech in 1992, for the first time, uses the concept of humanization of antibodies by replacing the CDR of mouse immunoglobulins with the corresponding human CDR sequence. For the first time, the patent US5750176A, filed by Abbot Laboratories in 1994, uses the concept of transgenic non-human mammals to produce recombinant enzymes and antibodies for better post-translational modifications with reduced immunogenicity. Another crucial patent, EP 0 590 058 B1, filed in 1992, focused on engineering humanized antibodies that bind specifically to the p185HER2 receptor. This patent was foundational in enabling the clinical-grade development of trastuzumab and supported consistent production for large-scale therapeutic use. Patients treated with this trastuzumab alongside chemotherapy showed improved survival and response rates, confirming its effectiveness [133].

The patent US005821.337A, published in 1998, provided detailed methods for producing monoclonal antibodies against p185HER2 using recombinant DNA and hybridoma technology [134]. This patent was crucial for developing HER2 diagnostic assays and reliable antibody production methods, essential for patient enrolment and stratification in clinical trials like NSABP B-31 and NCCTG N9831 using combination chemotherapy for HER2-positive tumours. This patent influenced the NSABP B-31 and NCCTG N9831 trials, where trastuzumab was combined with paclitaxel following doxorubicin and cyclophosphamide. These clinical trials aimed to find a difference in the efficacy of chemotherapy alone compared to trastuzumab + chemotherapy for HER2+ BC patients. These studies showed substantial improvements in disease-free and OS, highlighting the clinical relevance of combination therapy [135]. The HERA clinical trial (which started in 2001 and finished in 2015) analyzed the effect of trastuzumab therapy for 1–2 years for patients who had completed (neo-)adjuvant systemic chemotherapy, definitive surgery, and radiotherapy earlier, and the follow-up continued for 10–11 years. The results show that treatment with adjuvant trastuzumab for 1 year after chemotherapy, but not in combination, is associated with a significant clinical benefit over treatment alone. These patents and clinical trials form a tightly linked developmental arc for trastuzumab-based therapy for BC, where TR is not addressed.

## 10. Patents & Clinical Trials During the Period of Trastuzumab Resistance

John L. Bryant patented [136] the use of trastuzumab combined with at least one chemotherapeutic agent as adjuvant therapy to reduce recurrence after surgery and TR. This method demonstrated extended disease-free survival, establishing HER2-targeted antibodies as a cornerstone for preventing relapse. Due to its role in cancer progression, Wyeth LLC patented neratinib (a TKI) in 2010. The efficacy of neratinib was later evaluated in the NALA trial (NCT01808573) [137], a Phase III study comparing neratinib plus capecitabine versus lapatinib plus capecitabine in HER2-positive mBC patients previously treated with multiple HER2 therapies with possible relapse or TR. Neratinib improved PFS (8.8 vs. 6.6 months) and reduced CNS interventions, but it increased diarrhoea, highlighting the trade-off between efficacy and manageable toxicity.

Neratinib was selected as one of the agents for the I-SPY 2 trial, which began in 2010. The I-SPY 2 (Investigation of Serial Studies to Predict Your Therapeutic Response With Imaging And Molecular Analysis 2) trial (NCT01042379) is a neoadjuvant platform trial designed for newly diagnosed patients with locally advanced (Stage II/III) breast cancer, specifically those with tumor sizes of 2.5 cm or larger [138]. The I-SPY 2 trial, which involves immune checkpoint inhibitor pembrolizumab in combination with standard neoadjuvant chemotherapy, represents a landmark in trial design for oncology, utilising molecular biomarkers, imaging data, and adaptive methods to tailor therapy and enhance clinical research. Drugs such as Veliparib (PARP inhibitor) with Carboplatin, MK-2206 (AKT inhibitor), Ganitumab (IGF-1R inhibitor), Durvalumab (PD-L1 inhibitor) with Olaparib (PARP inhibitor) and Paclitaxel, and Trebananib were also evaluated in the I-SPY 2 trial as part of its adaptive, biomarker-driven neoadjuvant breast cancer study [139]. In 2012, Roche patented the triple combination of pertuzumab, trastuzumab, and carboplatin-based chemotherapy (including docetaxel) for neoadjuvant treatment of early-stage HER2-positive BC which are confined to the breast or axillary lymph nodes [140]. The landmark CLEOPATRA trial (NCT00567190) confirmed that adding pertuzumab to trastuzumab and docetaxel significantly increased median OS (56.5 vs. 40.8 months) and PFS, transforming first-line treatment for mBC for relapse cases [141]. In 2016, Daiichi Sankyo patented trastuzumab emtansine (T-DM1), an antibody–drug conjugate linking trastuzumab to the cytotoxic agent DM1, allowing targeted delivery of chemotherapy directly to HER2-overexpressing BC cells [142]. According to NCCN guidelines, the combination of pertuzumab, trastuzumab, and a taxane (such as docetaxel) is now the most common standard of care for patients who have not previously received any anti-HER2 therapy or chemotherapy for metastatic breast cancer (mBC) [143]. The EMILIA trial (NCT00829166) confirmed that T-DM1 significantly improved PFS (9.6 months) and OS (30.9 months) compared to lapatinib plus capecitabine in patients previously treated with trastuzumab and taxanes [144]. The trial also showed lower severe adverse events, underscoring T-DM1’s favourable safety profile. The results of the EMILIA trial established trastuzumab emtansine (T-DM1) as a standard second-line treatment according to NCCN guidelines for HER2-positive patients with locally advanced or metastatic breast cancer (mBC) who have previously received trastuzumab and taxane combination therapy [145]. The Incyte Corporation patented (US 20200399273A1, 2020) sulfonyl amide-based CDK2 inhibitors to restore sensitivity to trastuzumab by inhibiting CDK2 activity, which was involved in TR. For example, current ongoing trials (NCT04553133, NCT05252416) show the exclusive use of CDK2 inhibitors for clinical trials of metastatic breast cancer patients with relapsed cases. Further, the phase 1 trial has established the maximum tolerated dose for a CDK4/6 inhibitor for metastatic breast cancer patients in a clinical trial with TR.

To improve administration, Genentech patented a subcutaneous fixed-dose combination of pertuzumab, trastuzumab, and recombinant hyaluronidase to allow faster and more convenient delivery compared to intravenous infusions [146]. In the phase 3 study of the FeDeriCa trial, subcutaneous delivery showed the same pharmacokinetic properties and safety profile as intravenous delivery [79]. This improvement makes things easier for patients without making treatment less effective.

In parallel, the exploration of immunotherapy in HER2-positive BC led to the PANACEA trial (NCT02129556) [81], where they studied pembrolizumab, a PD-1 immune checkpoint inhibitor, with trastuzumab for trastuzumab resistance, and it showed high potential by showing 15% ORR.

The HER2CLIMB trial (NCT02614794) investigated tucatinib, a selective HER2 TKI, combined with trastuzumab and capecitabine in heavily pretreated metastatic HER2-positive BC, including patients with active brain metastases [147]. Tucatinib significantly improved PFS (7.8 vs. 5.6 months), OS (21.9 vs. 17.4 months), and delayed CNS disease progression, offering a new effective therapy for patients with CNS involvement, a population with limited options. Both trials have succeeded for relapsed cases, indicating their efficacy against TR.

Most recently, AstraZeneca have started a trial DESTINY-Breast09 (NCT04784715) exploring Trastuzumab Deruxtecan (T-DXd) with or without pertuzumab to see whether patients live longer or get worse compared to standard chemotherapy treatment. Probably at the end of 2029, we will learn how the treatment and cancer affect patients’ lives through this study [148].

**Figure 8:**
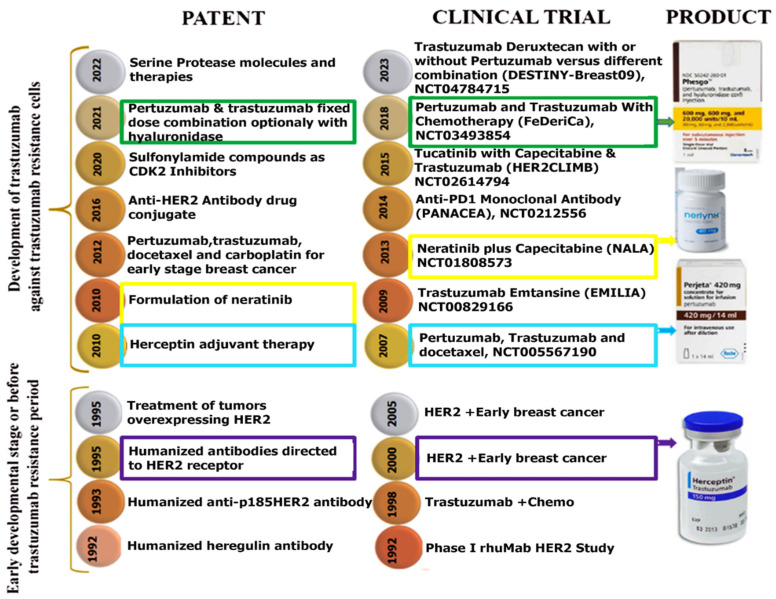
A brief timeline of pre-trastuzumab resistance and post-trastuzumab resistance periods with developed patents, and major clinical trials which led to a few marketed products of trastuzumab. Here, the rectangle with a similar colour code and arrow, together, shows the consequent progress of a patent to clinical trial to a marketed product.

Ongoing and already done clinical trial aims are to assess the ORR and safety for patients, which opens the treatment options for patients of different stages of disease. In addition, patents on innovative agents such as recombinant serine protease granzyme B polypeptides with enhanced stability have been filed to target lapatinib or trastuzumab-resistant cancer cells, demonstrating continued efforts to develop novel biologics against TR. Aside from these, in Figure 8, we attempted to outline relevant patents, clinical trials, and products established before and after the development of trastuzumab resistance.

## 11. Important Patents Identifying Novel Biomarkers Related to Trastuzumab Resistance

The patent filed by Jiangsu Cancer Prevention Research Institute, China, has shown the application of NDUFA4L2 (NADH dehydrogenase ubiquinone 1 alpha subcomplex, 4-like 2) in HER2+ BC resistance. The resistance of HER-2 positive BC towards trastuzumab can be reversed at the molecular level by NDUFA4L2 by creating a treatment-resistant stable cell strain of BC. Here, a complete genome study on the drug effect on Herceptin sensitivity or resistance in BC cells is performed, where the NDUFA4L2 gene was the most highly affected among 453 studied genes [Table 1]. They have claimed that NDUFA 4L2 can play a role in reversing a Herceptin-resistance of a HER2+ BC cell line BT474, where the level of NDUFA4L2 is highly overexpressed [149] [CN113308542]. Another application filed by the Jiangsu Cancer Hospital, China, is the role of UGT1A7 (UDP Glucuronosyltransferase Family 1 Member A7) for HER2-resistant BC. The invention shows that the expression of UGT1A7 is significantly lower in a Herceptin-resistant BT474HR cell than in the susceptible parent cell line. UGT1A7 expression is controlled via mitochondria and endoplasmic reticulum (ER) stress after trastuzumab treatment [150] [CN115029435]. Another invention using the CRISPR/Cas9 library claimed that inhibition of FGFR4 (fibroblast growth factor receptor 4) enhances the sensitivity of BC in HER2+ therapy. The mechanistic studies show that RNA m^6^A hypomethylation in BC cells upregulates FGFR4, which phosphorylates GSK-3β to stimulate a beta-catenin/TCF4 signal to drive the drug resistance to HER2 therapy. Furthermore, they have shown synergistic anti-HER2 and anti-FGFR4 antibody action in HER2+ BC therapy using the patient-derived xenograft and organoid susceptibility testing [149] [CN114736966].

Another invention (Seagen Inc., Paris, France) claimed that a mixture of tucatinib, capecitabine, and trastuzumab treatment lessens the consequences of HER2-positive BC. The patients experienced at least 7.5 months of PFS after the combination treatment [151] [WO2021080983]. The role of USP37 (Ubiquitin specific protease 37), a deubiquitination enzyme, as a drug target in drug-resistant BC was claimed in another invention [152] [CN113640518]. This discovery aims to ascertain whether the USP37 is involved in the progression of BC and whether targeting USP37 in conjunction with chemical-based (cisplatin) or radiation treatments will improve the efficacy of treatment for BC patients [Table 1]. A trinuclear platinum coordinating chemical derived from trimeprazine-based derivatives is disclosed in another invention [153] [CN113336798]. Trimeprazine is an antihistamine that is commonly used for allergies. In this case, the trimethoprim-based.

**Table 1:** Patent applications with major claims to target trastuzumab-resistant HER2+ BC.

Patent Application No. and Publication Year	Major Claims	Molecular Mechanism	Reference No.
CN113308542, 27.08.2021	1. A Trastuzumab-resistant cell BT474 HR is generated where protein NDUFA 4L2 is over-expressed significantly among 453 studied genes. 2. NDUFA 4L2 can be new target for studying TR	In herceptin drug-resistant BT474 HR cells, the expression of a protein, NDUFA 4L2, is significantly increased, and is mainly located in mitochondria of cells. The protein NDUFA 4L2 is identified as a new drug target in HER2+ BC cells.	[149]
CN112870193, 01.06.2021	1. A new composition for treating HER2-positive BC, composed of melatonin and a Tyrosine kinase inhibitor (TKI) lapatinib or neratinib 2. Both melatonin and Melatonin + TKI reduces the expression of HER2 receptor in sensitive and resistant cells	Melatonin synergizes the effect of small molecule TKIs in reducing the expression level of HER2 receptor in different HER2 positive and TR BC cells.	[75]
CN112316146, 05.02.2021	1. The expression of Ubiquitin carboxyl terminal hydrolase-L1 (UCH-L1) through a plasmid based expression in HER2 positive BC cells decreases the expression of HER2. 2. Co treatment of UCH-L1 plasmid + lapatinib kills the BC cells significantly by reducing the HER2 receptor expression	The expression of UCH-L1, which is a deubiquitinase changes the ubiquitination level of HER2 protein and make them more degradation prone thereby reducing the HER2 protein level in UCH-L1 over-expressing BC cells.	[154]
CN115029435 09.09.2022	1. UGT1A7 expression can reverse the HER-2 positive BC cells TR 2. UGT1A7 expression is drastically down-regulated in the BT474 resistant cell.	UGT1A7 expression is controlled via mitochondria and endoplasmic reticulum (ER) stress after trastuzumab treatment	[150]
CN114736966 12.07.2022	1. Using CRISPR/Cas9 library it was claimed that inhibition of FGFR4 (fibroblast growth factor receptor 4) enhances the sensitivity of BC in HER2+ therapy 2. Synergistic action of anti-HER2 and anti-FGFR4 antibody in HER2+ BC therapy was shown	RNA m^6^A hypomethylation in BC cells upregulates FGFR4, which phosphorylates GSK-3β. It stimulate a beta-catenin/TCF4 signal to drive the drug resistance to HER2 therapy.	[149]
CN113640518 12.11.2021	1. USP37, a new protein, was found to be involved in the progression of BC. 2. Combination of USP37+ cisplatin or radiation treatments will improve the efficacy of treatment for BC patients	The deubiquitinating enzyme USP37 kills BC more than cisplatin does because the cells become more degradation prone after USP37 expression	[152]

Trinuclear platinum coordination compounds exhibit an IC_50_ of 3.6–18 µM against the BC cells. The invention claims the first trinuclear derivative of cisplatin for the application of BC. In another patent, the combination therapy of trastuzumab and cold atmospheric plasma (CAP) was claimed as an effective therapy for HER2-positive and HER2-negative BC patients with [155] [US20200069958]. This is based on previous observations that CAP works very well for wound healing in clinical trials. The US patent WO2019098682 claims the discovery of a new antibody (a humanized Mab with scFv) against HER2, which is more effective against HER2+ BC than trastuzumab because the new antibody targets a new domain on HER2 that is different from trastuzumab [156] [WO2019098682].

The patent of Xiangya Hospital, China, claims a new biomarker, UCH-L1 (Ubiquitin C-terminal hydrolase L1), which is under-expressed in HER2+ BCs. UCH-L1 [Table 1] is a deubiquitinase that controls the proteasomal degradation of proteins (more commonly for neuronal proteins) by removing Lys48-linked polyubiquitin chains. They claim that UCH-L1 can be used as a drug target alone or in combination with trastuzumab for HER2+ BC, since it inhibits the expression of HER2 protein in BC [154] [CN112316146]. In another invention, a nanoparticle composed of a peptide, nucleic acid, and doxorubicin (which are formed by non-covalent and anionic interactions) was used for the treatment of a patient who is not responding to trastuzumab. The techniques for treating cancer in patients are described, which target the HER3 receptors [157] [EP3129066]. Here, the peptide NP targets the HER3 receptor, where, after endocytosis, the doxorubicin is released to treat the BC.

The potential use of a hormone therapy using melatonin to treat HER2-positive BC is described in an invention. Melatonin is a hormone that is produced by the brain in darkness and controls our circadian rhythm. Studies demonstrate that melatonin can help treat HER2-positive BC by modifying the structural integrity of the HER2 protein. They also demonstrate that melatonin can improve the therapeutic impact of HER2 small-molecule targeted drugs (for example, lapatinib and neratinib, which is used in this study) on HER2-positive BC that is resistant to these drugs, and that melatonin could be used to stop HER2-positive BC metastasis and recurrence [75] [CN112870193]. A therapeutic approach for HER2+ and trastuzumab-resistant BC was claimed by studying the expression level of Mucin 4 protein in those patients. Here, a combination therapy including a selective inhibitor of soluble TNFα and trastuzumab is used for those patients where the level of Mucin-4 is greater than a threshold level [158] [US20200147175]. These unconventional biomarkers/pathways, which are claimed in the patents, have not been tested yet in any of the clinical trials. However, looking at the success of combination therapy for HER2+ BC, it is likely that therapeutic approaches involving these pathways may be explored very soon to tackle TR.

## 12. Conclusions and Future Directions

It is important to note that in only 20% of BC, the HER2 is overexpressed, whereas the other forms of BC can be due to other factors like angiogenesis via VEGF, cytokine imbalance, chronic inflammation, and changes in TME or oxidative stress, etc. The plasma and serum levels of VEGF are important biomarkers for BC, as it was found that serum and plasma levels of VEGF are higher in mBC than in healthy volunteers [159]. The C-reactive protein (CRP), considered a classic marker for chronic inflammation, was identified in nipple aspirate fluid of healthy women and has been positively correlated to BC risk [160]. The crosstalk of inflammatory cytokines in the TME of BC has identified a few stimulatory cytokines (leptin, IL-1β, IL-6, IL-8, etc.) or inhibitory (IL-2, IL-12, and IFN-γ) for BC [161]. Higher levels of 8-hydroxy-2′-deoxyguanosine, a common biomarker for ROS-mediated DNA damage, are consistently higher in BCs than in normal cells [162]. This indicates that higher ROS can cause BC. Although all these factors are related to HER2-negative BC, it will be interesting to see the fate of these factors when HER2+ BCs are treated with trastuzumab and/or chemotherapy.

Based on the current market sales, the top 3 Mabs are Humira (adalimumab, which targets TNF-α), Keytruda (pembrolizumab, which binds PD-1), and Stelara (Ustekinumab, which targets IL-12 and IL-23). Among these, Humira and its biosimilars are used to treat various inflammatory diseases, including juvenile idiopathic arthritis, Crohn’s disease (CD), plaque psoriasis, rheumatoid arthritis (RA), psoriatic arthritis (PsA), etc. This possibly explains its demand and current market sales [163]. Among the top 20 mAb sales, trastuzumab does not come into the picture, and the mAb, pertuzumab, which acts against HER2+ BC like trastuzumab, is in 11th position with market sales of USD4.4B till 2022 [105]. Nevertheless, pertuzumab binds to domain II of HER2, whereas trastuzumab binds to domain IV. Further, pertuzumab efficiently inhibits ligand-induced HER2/HER3 dimerization, whereas trastuzumab’s significant action is ADCC. The decrease in sales indicates that resistance against trastuzumab is a serious issue, and it is thereby replaced by other antibodies or in combination therapy, where trastuzumab is always combined with other Mab, chemotherapy, and/or tyrosine kinase inhibitors. Based on current patents, the ubiquitin proteosome system was identified as a possible target for HER2+ BC treatment. However, to date, no studies have been carried out using proteosome inhibitors in combination with trastuzumab for overcoming HER2+ BC in a clinical setting.

Two Mabs that act as immune checkpoint inhibitors (Pembrolizumab and Atezolizumab) are combined with Trastuzumab or T-DM1 (ADC of trastuzumab) in clinical trials to treat the TNBC or HER2+ BC, where regular trastuzumab therapy fails [164]. The success of these studies indicates that trastuzumab alone is not recommended for any HER2+ BC therapy because patients generate resistance through different pathways (eg, AKT/PI3K/mTOR, NF-κB, notch signalling, etc.) within 1 year of treatment. Most combination therapy includes TKIs, among which lapatinib, neratinib, and pyrotinib are extensively used, because of several advantages, including oral administration, reduced cardiotoxicity, and favourable blood–brain barrier penetration in BC. Apart from combination therapy, ADCs like Trastuzumab emtansine (T-DM1) and trastuzumab deruxtecan (T-DXd) are being used for resistance against HER2+ BC, but the success is not better than trastuzumab alone [165]. TR in HER2+ BC overcoming possibly requires a modified Fc-based Trastuzumab with improved ADCC or a better chemotherapeutic agent apart from TKIs, which can be combined with trastuzumab [166].

## Data Availability

No new data were created or analyzed in this study. Data sharing is not applicable to this article.

## Abbreviations

BC	Breast Cancer
BCSC	BC stem cell
TR	Trastuzumab Resistance
ADC	Antibody drug conjugate
ADCC	Antibody-directed cellular cytotoxicity
HER2	human epidermal growth factor receptor 2
EGFR	Epidermal growth factor receptor
ErBb	Erythroblastic oncogene B
ER	Estrogen receptor
PR	Progesterone receptor
PD-L1	Programmed Death Ligand 1
PD-1	programmed death 1
PFS	progression free survival
OS	overall survival
ORR	overall response rate
MUC1	Mucin 1
PI3K	Phosphatidyl Inositol 3 Kinase
mTOR	Mammalian target of rapamycin
MAP2K1	Mitogen-activated protein kinase kinase 1
TME	Tumour Micro Environment
TKI	Tyrosine kinase inhibitor
TNBC	Triple negative BC
IGF-1R	Insulin-like Growth Factor 1 receptor
MET	Mesenchymal epithelial transition
NR2F2	Nuclear Receptor Subfamily 2 Group F Member 2
CAR	Chimeric Antigenic Receptor
CEA	Carcinoembryonic Antigen
CXCL2	C-X-C motif chemokine ligand 2
CCND1	Cyclin D1
STAT3	Signal transducer and activator of transcription 3
SLC38A1	Solute Carrier Family 38, Member 1
TGF-β	Transforming growth factor β
